# Normative tonsillectomy outcome inventory 14 values as a decision-making tool for tonsillectomy

**DOI:** 10.1007/s00405-020-06374-0

**Published:** 2020-09-22

**Authors:** Michaela Plath, Matthias Sand, Philippe A. Federspil, Peter K. Plinkert, Ingo Baumann, Karim Zaoui

**Affiliations:** 1 Department of Otorhinolaryngology, Head and Neck Surgery, University Hospital Heidelberg, Ruprecht-Karls-University, Im Neuenheimer Feld 400, 69120 Heidelberg, Germany; 2 GESIS-Leibniz-Institute for the Social Sciences, Mannheim, Germany

**Keywords:** TOI-14, Quality of life, Recurrent tonsillitis, Guidelines, PCA, EFA, Middle-european cohort

## Abstract

**Purpose:**

The study aimed to determine normative values for the Tonsillectomy Outcome Inventory 14 (TOI-14) in a healthy middle-European cohort. We also compared these generated values with TOI-14 scores from a patient population with recurrent tonsillitis (RT) and explored the factorial structure of the TOI-14.

**Methods:**

We systematically studied the responses of healthy individuals (reference cohort) and patients with RT (clinical cohort) to the TOI-14 survey. The reference cohort contained 1000 participants, who were recruited using the Respondi panel for market and social science research. This subsample was quoted to the population distribution of the German Microcensus and selected from a non-probability panel. Tonsillitis patients were assessed before and 6 and 12 months after tonsillectomy.

Data were analysed using principal component and exploratory factor analyses.

**Results:**

The PCA revealed three TOI-14 domains (physiological, psychological and socio-economic), which explained 73% of the total variance. The reference cohort perceived a good quality of life (QOL) with a TOI-14 total score of 11.8 (physiological: 8.0, psychological: 5.8, and socio-economic subscale score: 13.9). TOI-14 scores were higher in the patient cohort, indicating that the TOI-14 discriminates between patients with RT and healthy individuals with no RT. Age and female gender significantly influenced the total TOI-14 score, especially in the psychological (age) and socio-economic (gender) subscales.

**Conclusion:**

We have developed a set of normative values that, together with the TOI-14, can determine the disease burden indicating tonsillectomy.

## Introduction

Tonsillectomy is one of the most common surgical interventions [1, 2]. The main indications for tonsillectomy are recurrent infections, suspicion of malignant disease, and grade four tonsillar hypertrophy (kissing tonsil) with obstructive sleep apnoea [3]. Recurrent tonsillitis (RT) reduces the disease-specific quality of life (QOL) [2]. The effectiveness of tonsillectomy as a treatment for RT in adults is controversial. Previous studies have shown that tonsillectomy is associated with improved health-related quality of life (HR-QOL) [1, 2, 4, 5], reduced medication consumption, less time off work, and fewer visits to the physician [6]. In another study, 75% of tonsillectomies for RT resulted in postoperative pharyngitis and upper respiratory tract infections [7].

In 2013, the Bertelsmann Foundation reported that the frequency of tonsillectomy differed regionally in Germany by a factor of eight. To address this problem, guidelines for and classification of conservative and surgical treatments were developed. In August 2015, the AWMF (a consortium of scientific medical societies) published the new S2k guidelines on the therapy of palatine tonsillitis. A main focus of the new guidelines was defining the indications for tonsillectomy. The guidelines specified that a tonsillectomy should only be considered if purulent tonsillitis has been treated with antibiotics six times within the past 12 months [8].

The lifetime prevalence of common RT is 7–11% [9]. Only a few studies have addressed whether recurrent tonsillopharyngitis affects QOL in adults [1, 5, 6, 10, 11], although this has been well-studied in children [6, 7, 12–24]. In 2012, Skevas et al. introduced the Tonsillectomy Outcome Inventory 14 (TOI-14), which was the first worldwide-validated instrument to measure disease-specific QOL in adults with RT [25]. However, the TOI-14 is not yet commonly used in clinical practice to decide whether tonsillectomy is necessary in cases of RT.

In the present systematic prospective study, TOI-14 scores were measured in two cohorts: a middle-European reference cohort of 1000 healthy individuals and a clinical cohort of 108 tonsillitis patients. The healthy volunteers were recruited from a non-probability panel. The subsample has been quoted to the population distribution of the German Microcensus with respect to age, gender, education and region. The tonsillitis patients were scheduled for elective tonsillectomy in our department. We compared the HR-QOL of healthy individuals and tonsillitis patients, examined the factorial structure of the TOI-14 questionnaire using principal component analyses (PCA) and exploratory factor analyses (EFA), and explored whether normative TOI-14 scores can define the level of disease burden that justifies tonsillectomy.

## Material and methods

### Recruitment and patient data

Our target collective corresponds to a non-probabilistic quota sample (*n* = 1000), which was quoted for the German population by Microcensus, a 1% probability sample of the German population that is repeated annually and for which the participation is required by law [26]. Relevant parameters were age, gender, region and education. The average age was 44.3 ± 14.2 years. Healthy participants were recruited by September 2018 using the Respondi panel, an international organization for standardization (ISO)-certified online access panel for market and social science research in Europe. All patients were informed about the study aims and protocol, and participants were enrolled after giving informed written consent.

Data were also collected from a clinical cohort of patients with RT, who were scheduled for elective tonsillectomy at the Department of Otorhinolaryngology, Head and Neck Surgery at the University Hospital Heidelberg, Germany. The Ethics Committee of the Medical Faculty at the University of Heidelberg granted permission to conduct the study (Project No. 363/2005) according to the Declaration of Helsinki on biomedical research involving human subjects. RT patients were assessed before and 6 and 12 months after elective tonsillectomy. The patient cohort contained 108 individuals before tonsillectomy [25], 58 patients 6 months after tonsillectomy, and 42 patients 12 months after tonsillectomy. The data lacks the reasons for patients dropping out from one point in time to another.

### TOI-14 questionnaire

TOI-14 is a reliable disease-specific questionnaire that was validated in Germany by Skevas et al. The questionnaire was systematically developed from an initial set of 28 questions (TOI-28 alpha) based on a literature search for tonsillitis symptoms and their effects on QOL symptoms [27]. Questions were answered according to a six-point Likert scale with scores ranging from zero (no problem) to five (worst possible). Points were added up, divided by the number of questions, and multiplied by 100 to get subscale scores and total scores. Scores ranged from 0 to 100, with higher scores indicating a higher disease burden [10].

### Statistical analysis

Statistical analysis was performed by one of the authors as a certified expert of survey analysis, who is working at the GESIS-Leibniz-Institute for the Social Sciences. Data were analysed using the statistical software R (version 3.5.2). PCA and EFA were conducted using *psych, nFactor, and FactoMineR* libraries. To determine the number of main components, graphical and non-graphical PCAs were used, including scree plots and the analyses of the models eigenvalues, parallel analysis and the determination of optimal coordinates as suggested by Kaiser [28] and Cattell [29]. The TOI-14 questionnaire stems from a non-probability survey. So, data analysis was mostly descriptive because the non-probabilistic approach prohibited any inference. However, it can be assumed that the underlying data-generating process is not dependent on the measured indices, giving us the first insights into a “healthy cohort”. PCA and EFA indicated that the TOI-14 score can be explained by three components or sub-indices that can each be described by four to five different variables. These sub-indices were calculated for each individual within the data set, rescaling its value to the range of the original TOI-14 for better comparison. The results of each sub-index were then compared with the overall TOI-14 score.

Socio-demographic characteristics were analysed using generalized regression models. Metric variables are presented as means ± standard deviation, and categorical variables are presented as absolute numbers and percentages. Potential differences between groups were examined using the Wilcoxon’s test for nonparametric data and Student’s test for parametric data. Differences in TOI-14 scores between groups were determined using paired *t *test and one-way ANOVA. A *p*-value less than 0.05 was considered statistically significant. Data from the healthy reference cohort and the patient cohort were compared. All sub-indices were measured in the patient cohort before tonsillectomy and 6 and 12 months after tonsillectomy and were compared with the healthy reference cohort using the *ggplot2* package of R (Fig. 3).

## Results

### PCA and EFA analysis

**Fig. 1:**
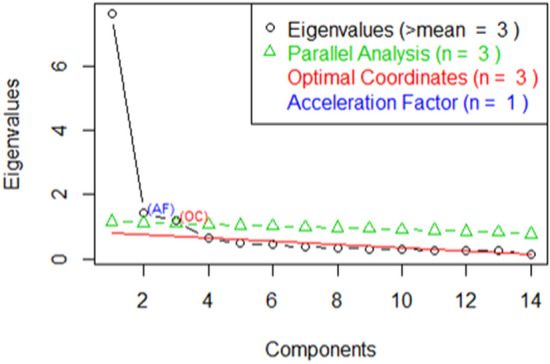
Non-graphical analysis as elbow figure. The black curve, illustrating the Eigenvalues in descending order, would suggest the inclusion of three main components since any further Eigenvalues are below one. However, the parallel analysis (triangles upward), comparing the actual matrix of Eigenvalues to a Monte-Carlo-simulated matrix of the same size, shows that only the EVs of two main-components are above the 95th percentile. The same number of components is suggested in regard to the EVs’ gradients and the optimal coordinates (triangles downward), whereas the Acceleration Factor

According to the subscores of Skevas et al. [25], we intended to reduce methodologically the 14 symptoms into umbrella terms using PCA. The graphical and non-graphical analyses (Fig. 1) revealed three components—physiological, psychological, and socio-economic—which explained 73% of the total variance. All factor loadings were > 0.5. Next, we calculated the EFA for these three components (Table 1). Question 12 (“reduced participation”; 0.82) and question 13 (“fewer gatherings”; 0.81) fitted best into the psychological domain; question 3 (“sore throat”; 0.69) fitted best into the physiological domain; and question 10 (“cost of medications”; 0.73) fitted best into the socio-economic domain.

**Table 1:** TOI-14 questionnaire is split into three categories

TOI-14 questionnaire	Psychological	Physiological	Socio-economic
1. Dry throat		0.66	
2. Thick secretion (catarrh) in the throat		0.66	
3. Sore throat	0.38	0.69	
4. Swallowing difficulties	0.39	0.67	
5. Feeling ill		0.63	0.50
6. Reduced ability to work or do daily chores		0.46	0.60
7. Frequency of visits to the doctor		0.35	0.72
8. Cost of doctor visits (missing work, travel, parking, etc.)			0.69
9. Frequency of antibiotics use	0.45		0.55
10. Costs of medicine (prescription or over-the-counter)			0.73
11. Trouble at work as a result of missing working days because of tonsillitis/sore throat	0.72		
12. Reduced participation in events/activities as a result of tonsillitis/sore throat	0.82	0.34	
13. Fewer gatherings with family/friends as a result of tonsillitis/sore throat	0.81	0.31	
14. Feeling depressed as a result of tonsillitis/sore throat	0.75	0.31	

Depending on the higher loading, the category was assigned to the respective factor 1 (psychological), factor 2 (physiological), or factor 3 (socio-economic) according to explorative factor analysis

### Comparison with tonsillitis patients

To determine whether the TOI-14 discriminates between healthy individuals without chronic throat problems and patients with chronic tonsillitis before and after tonsillectomy, we compared data between the reference and clinical cohorts [25]. Tonsillitis patients (*n* = 108) had higher TOI-14 scores than healthy individuals before tonsillectomy (52.3 versus 11.8; *p* < 0.01), 6 months after tonsillectomy (*n* = 58; 53 versus 11.8; *p* < 0.01), and 12 months after tonsillectomy (*n* = 42; 52.48 versus 11.8; *p* < 0.01), indicating higher levels of disease burden. The high drop-out rate after the first wave (pre-tonsillectomy), leading to a smaller sample size, may hereby have negatively impacted the estimators’ variance. To mitigate such problems, we opted for a variance estimation based on Monte-Carlo-Simulations for our comparisons. Nevertheless, since the Monte-Carlo-Variance is also an estimate and cannot reduce the impact of a small sample size in its entirety, the generalizability of these results is limited. Due to the increased variance, the probability of false-negative results (no difference, when there actually is any) may be increased. However, results that show a significant difference in estimates may be expected to remain so, if the sample size would have been larger.

**Fig. 2:**
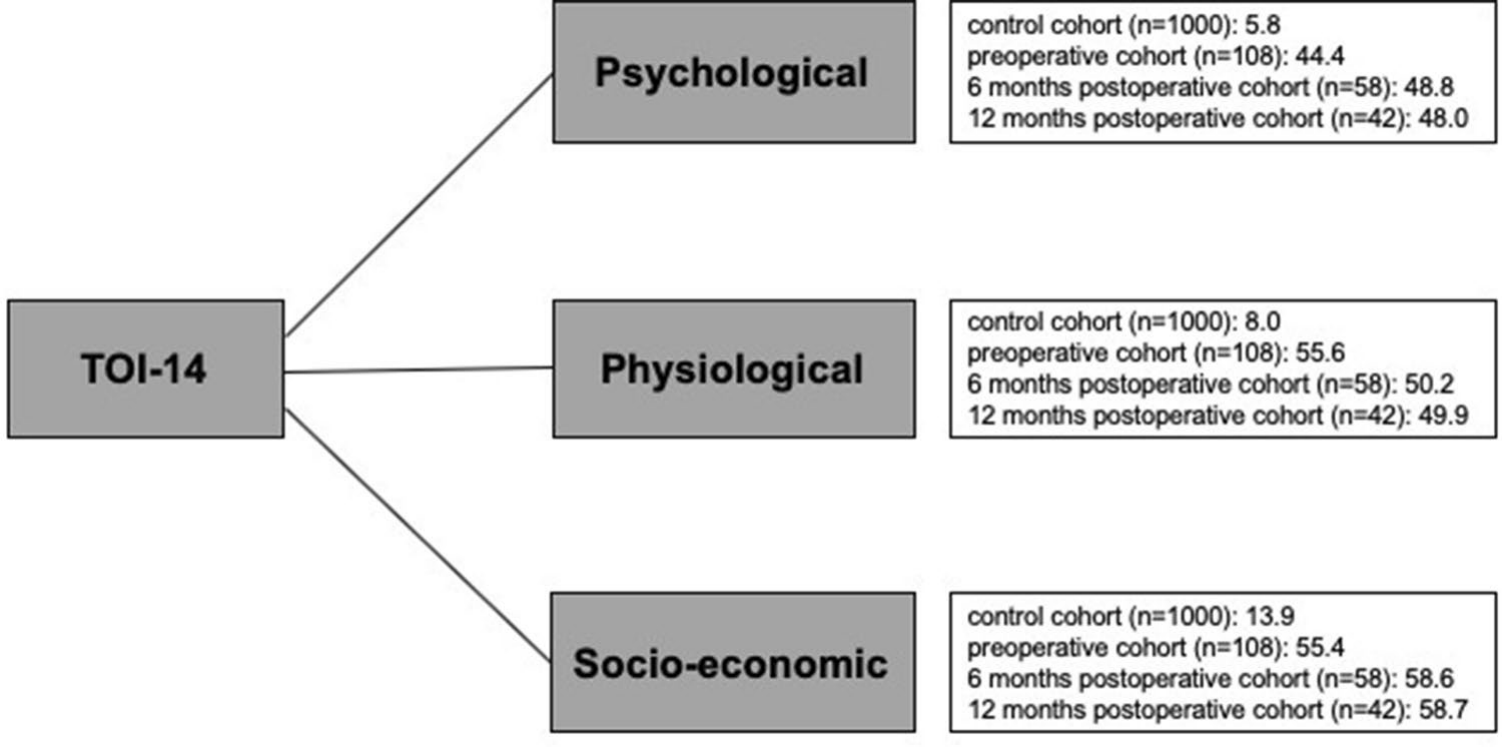
An overview of TOI-14 sub-indices of the four different cohorts (control, preoperative, 6 months postoperative and 12 months postoperative) independent of the time point

**Fig. 3:**
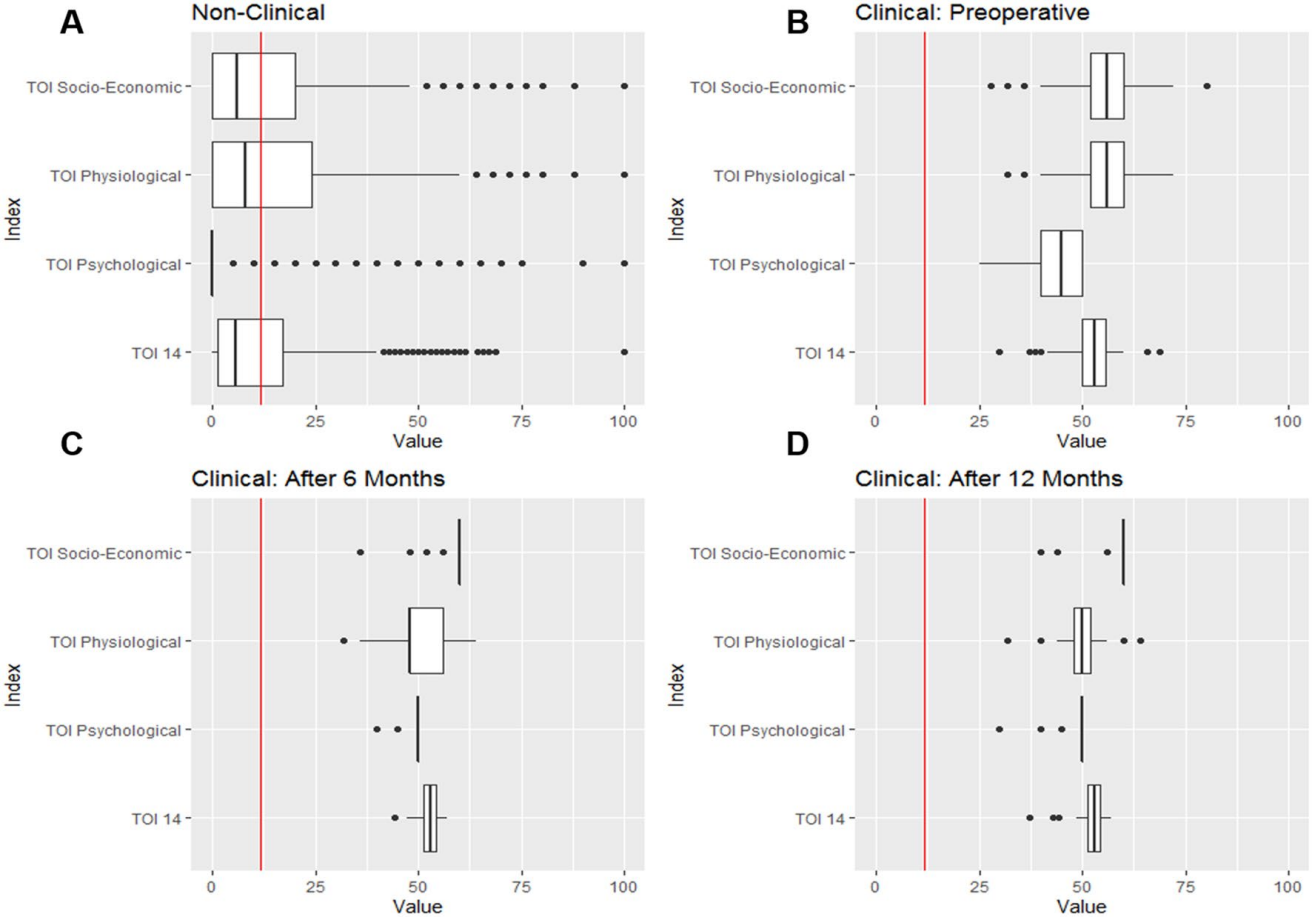
Boxplot illustration of the TOI-14 overall score (bottom) and the novel TOI-14 sub-indices of the reference cohort (**a** *n* = 1000), the preoperative patient cohort (**b** *n* = 108), the 6-month postoperative patient cohort (**c** *n* = 58), and the 12-month postoperative patient cohort (**d** *n* = 42). The red line represents the overall mean TOI-14 score of the non-clinical cohort (11.8). The bold line describes each distribution’s median whereas the box represents the interquartile range. Dots resemble outliers

TOI-14 scores were not significantly different after tonsillectomy; this lack of difference may be explained by the high dropout rate in this already small sample. Creating TOI-14 sub-indices determined by PCA and EFA revealed differences in TOI-14 sub-index values before and after tonsillectomy: psychological and socio-economic scores were higher after tonsillectomy (psychological: control = 5.8; 6 months = 48.8, 12 months = 48.0; socio-economic: control = 13.9; 6 months = 58.6, 12 months = 58.7) whereas physiological scores were higher before tonsillectomy (control = 8.0; preoperative = 55.6; 6 months = 50.2; 12 months = 49.9) (Fig. 2). As expected, the healthy cohort had the lowest scores. Interestingly, although all sub-index scores were significantly lower in the healthy cohort, the variation between scores was larger in this group (Fig. 3b–d).

### Influence of socio-demographic aspects

**Fig. 4:**
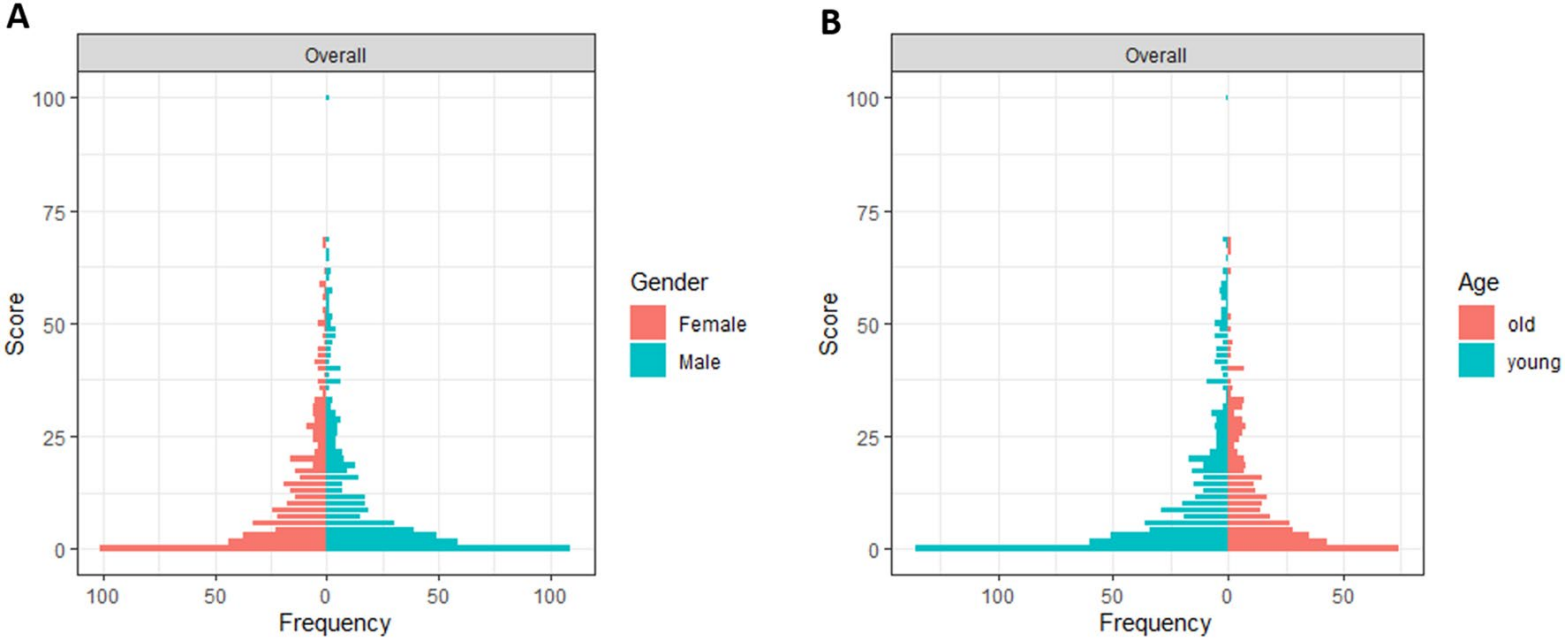
Forest plot showing the association between overall TOI-14 score and gender (**a**) and age (**b**) of the reference cohort (*n* = 1000). The questionnaire score is plotted on the *Y*-axis, the frequency of score responses on the *X*-axis

Regarding socio-demographic aspects, only age ($\beta$= − 0.1, *p* = 0.03) and female gender ($\beta$= 1.92, *p* = 0.05) significantly affected TOI-14 scores in the reference cohort (*n* = 1000; average TOI-14 score = 11.8). TOI-14 scores did not differ significantly between males (11.12, *n* = 500) and females (12.43, *n* = 500) according to the paired *t* test (*p* = 0.16), whereas scores were significantly higher (12.50 versus 10.68; *p* = 0.04) in older (> 50 years, *n* = 400) participants than younger participants (< 50 years; *n* = 600) (Fig. 4). However, although the impact of age was significant, the beta-coefficient was low (*ß* = − 0.1).

Physiological sub-index scores were not significantly affected by socio-demographic factors. Psychological sub-index scores were affected by age (*p* < 0.01) and socio-economic sub-index scores were affected by female gender (*p* = 0.02): psychological scores were lower in older individuals while socio-economic scores were higher in female participants.

## Discussion

Quality of life (QOL) measurements are becoming more important in otolaryngology [27], especially in the case of tonsillectomy, which is a common otolaryngological operation [6]. Studies have focused on the outcome and benefit of tonsillectomy in children with obstructive sleep apnoea but studies in adult patients are still lacking. According to the published literature, tonsillectomy does not only impact QOL in children but may also affect the outcome in adults [1, 5, 6, 10, 11]. Measuring the success of a medical intervention requires analysis of patient-related outcomes. To date, only two disease-specific questionnaires relating to tonsillectomy and tonsillotomy have been validated for adult patients: the Tonsillectomy Outcome Inventory 14 (TOI-14) and the Tonsil and Adenoid Health Status Instrument (TAHSI). Adenoiditis is a paediatric disease, so the TAHSI questionnaire [10] cannot be used to assess QOL in adults with recurrent tonsillitis (RT). To determine whether patients with RT are suitable for tonsillectomy, standardized values from a sufficiently large healthy reference cohort need to be obtained from a tonsillitis-specific instrument. To address this, we measured TOI-14 scores in 1000 healthy volunteers and found that this reference cohort perceived a good QOL without RT.

To our knowledge, only a few studies have assessed the TOI-14 score before elective tonsillectomy. In the first study, suitability for tonsillectomy was determined by the SIGN 117 guidelines, and 97% of the preoperative TOI-14 scores were within two standard deviations (range 26.22–65.02; mean 45.62) [27]. Skevas et al. measured TOI-14 scores before and after tonsillectomy and compared these scores to those from 67 healthy individuals, but this study had insufficient power [25]. This study also evaluated TOI-14 subscales: throat problems (questions 1–4), overall health (questions 5–6), resources (questions 7–10), and social-psychological restrictions (questions 11–14) [25]. However, the test–retest-reliabilities of the subscales “general health” (*r* = 0.45) and “resources” (0.44) were only moderate. To address this, we reperformed PCA and EFA and proposed three novel TOI-14 subscales—physiological (question 1–5), psychological (questions 11–14), and socio-economic (questions 6–10)—which explained 73% of the variance in TOI-14 scores. We did choose different umbrella terms than Skevas et al. [25] because our new PCA and EFA revealed novel assignments of items to particular components (as shown in Table 1), which is more plausible. Furthermore, general headings are easier to understand.

We also observed that questions 12 and 13 were relevant to the psychological impact of chronic throat problems, question 3 to the physiological impact, and question 10 impacted the socio-economic measurement. These findings indicate that the different TOI-14 subscale scores should be considered when deciding whether tonsillectomy would give the best patient outcome.

Concerning the socio-demographic aspects, age and female gender significantly influenced the TOI-14 score, especially in the psychological (age) and socio-economic (gender) subscales. This suggests that young and female patients with RT could benefit the most from tonsillectomy.

Our comparisons between the healthy reference cohort and clinical cohort of tonsillitis patients confirmed that the adverse effects of RT have a huge impact on disease-specific QOL in adults. Our preoperative patient cohort had much higher TOI-14 scores (with less variation) than the healthy reference cohort, indicating higher levels of disease burden. Absence from work and lack of concentration affected productivity and consequently the socio-economic status. This may lead to job insecurity, which negatively affects QOL and health [30]. Interestingly, TOI-14 scores were still higher in the postoperative patient cohorts than in the healthy cohort. The postoperative cohorts had the highest psychological and socio-economic TOI-14 sub-index values, and the preoperative cohort had the highest physiological values. These results raise the question as to whether adult patients really benefit psychologically and socio-economically from tonsillectomy.

The lack of benefits after tonsillectomy suggests that stricter indication criteria are needed. We started measuring our patient TOI-14 scores in 2012. Since then, the indications for tonsillectomy have been tightened thanks to the 2015 AWMF guidelines. Now, tonsillectomy should only be performed after six cases of purulent tonsillitis have been treated with antibiotics within the past 12 months. Our postoperative data need to be interpreted with caution because the drop-out rate was high, indicating potential bias.

A QOL assessment tool is needed that encompassed functional, psychological, and socio-economic properties of patients undergoing tonsillectomy. Close examination of our novel TOI-14 sub-index scores may help to select those patients who will benefit most from tonsillectomy.

## Conclusion

We have developed a set of reference values that, together with the TOI-14, can determine the disease burden that indicates tonsillectomy, according to the AWMF guidelines.

## Data Availability

The datasets generated during and/or analysed during the current study are available from the corresponding author on reasonable request.
